# Identification of radiation-induced aberrant hypomethylation in colon cancer

**DOI:** 10.1186/s12864-015-1229-6

**Published:** 2015-02-06

**Authors:** Jin-Han Bae, Joong-Gook Kim, Kyu Heo, Kwangmo Yang, Tae-Oh Kim, Joo Mi Yi

**Affiliations:** Research Center, Dongnam Institute of Radiological & Medical Sciences (DIRAMS), Busan, 619-953 South Korea; Department of Radiation Oncology, Korea Institute of Radiological and Medical Sciences, Seoul, 139-709 Korea; Department of Internal Medicine, Inje University Haeundae Paik hospital, Busan, 612-896 South Korea

**Keywords:** Ionizing radiation, 5-aza-2′-deoxycytidine, DNA hypomethylation, Methylation profiling, Gene ontology, Colon cancer

## Abstract

**Background:**

Exposure to ionizing radiation (IR) results in the simultaneous activation or downregulation of multiple signaling pathways that play critical roles in cell type-specific control of survival or death. IR is a well-known genotoxic agent and human carcinogen that induces cellular damage through direct and indirect mechanisms. However, its impact on epigenetic mechanisms has not been elucidated, and more specifically, little information is available regarding genome-wide DNA methylation changes in cancer cells after IR exposure. Recently, genome-wide DNA methylation profiling technology using the Illumina HumanMethylation450K platform has emerged that allows us to query >450,000 loci within the genome. This improved technology is capable of identifying genome-wide DNA methylation changes in CpG islands and other CpG island-associated regions.

**Results:**

In this study, we employed this technology to test the hypothesis that exposure to IR not only induces differential DNA methylation patterns at a genome-wide level, but also results in locus- and gene-specific DNA methylation changes. We screened for differential DNA methylation changes in colorectal cancer cells after IR exposure with 2 and 5 Gy. Twenty-nine genes showed radiation-induced hypomethylation in colon cancer cells, and of those, seven genes showed a corresponding increase in gene expression by reverse transcriptase polymerase chain reaction (RT-PCR). In addition, we performed chromatin immunoprecipitation (ChIP) to confirm that the DNA-methyltransferase 1 (DNMT1) level associated with the promoter regions of these genes correlated with their methylation level and gene expression changes. Finally, we used a gene ontology (GO) database to show that a handful of hypomethylated genes induced by IR are associated with a variety of biological pathways related to cancer.

**Conclusion:**

We identified alterations in global DNA methylation patterns and hypomethylation at specific cancer-related genes following IR exposure, which suggests that radiation exposure plays a critical role in conferring epigenetic alterations in cancer.

**Electronic supplementary material:**

The online version of this article (doi:10.1186/s12864-015-1229-6) contains supplementary material, which is available to authorized users.

## Background

Epigenetics is defined as heritable changes in gene expression that are not accompanied by changes in DNA sequences [1]. Epigenetic mechanisms are essential for the maintenance of tissue-specific gene expression patterns, and they are required for normal mammalian development. Disregulation of epigenetic processes can lead to altered gene function and malignant cellular transformation, and global changes in the epigenetic landscape are a hallmark of cancer.

Epigenetics is becoming more widely investigated in the field of cancer biology. Epigenetic regulation of gene expression is mediated primarily through DNA methylation, microRNAs (miRNAs), and histone modifications, such as acetylation, methylation, phosphorylation, and ubiquitination. DNA methylation is perhaps the most extensively studied epigenetic modification in mammals. Generally, there are two types of alterations that can occur in the DNA methylation patterns in cancer at both locus-specific and genome-wide levels: hypomethylation and hypermethylation [2]. In the field of cancer epigenetics, most research has focused on the role and clinical relevance of the hypermethylation of tumor suppressor gene promoters [3]. However, there have been far fewer studies on the role of DNA hypomethylation, even though global DNA hypomethylation is a well-known characteristic of cancer cells. This hypomethylation is specifically enriched in mobile genetic elements, such as long interspersed element 1 (*LINE1*) and *Alu* elements. [4].

Unlike genetic alterations, epigenetic alterations are often reversible, which makes elucidating the mechanisms of epigenetic regulation extremely attractive from the perspective of developing new therapeutic approaches. The reversible nature of epigenetic aberrations has led to the emergence of the promising field of epigenetic therapy.

Ionizing radiation (IR) is a potentially lethal threat to all organisms, but it is also an efficient and valuable treatment option in cancer therapy [5]. Radiation therapy triggers a complex, multi-pathway cellular response that usually results in the death of cancer cells. Although IR is a well-known genotoxic agent and human carcinogen that induces cellular damage through direct and indirect mechanisms [6], its impact on epigenetic mechanisms has not been elucidated.

Our previous study suggested that the combination of IR and the demethylating agent 5-aza--2′-deoxycytidine (5-aza-dC) could be an effective treatment strategy for colon cancer [7]. These data also prompted us to explore the potential molecular alterations responsible for the biological effects observed after treatment with IR and 5-aza-dC. In rodents, IR can affect global DNA methylation and cause significant and persistent DNA hypomethylation in radiation target genes, and in human cells, IR can cause genome instability [8]. However, there has been a lack of studies on the effects of IR on locus- and gene-specific DNA methylation. Several groups have performed gene expression profiling to understand how IR affects radiosensitive cancer cells at the molecular level [9,10]. Other studies have shown that treatment with 5-aza-dC to reduce global DNA methylation has a radiosensitizing effect, suggesting that DNA methylation plays a role in radiation resistance [11,12]. However, little information is available regarding DNA methylation changes at the genome-wide level in cancer cells after IR exposure.

Genome-wide DNA methylation profiling technology using the Illumina HumanMethylation450K array allows us to query >450,000 loci within the genome and to cover 99% of RefSeq genes [13]. This improved technology can provide a more powerful and comprehensive analysis of DNA methylation changes. In this study, we employed this technology to test the hypothesis that exposure to IR not only induces differential DNA methylation patterns at a genome-wide level, but also affects methylation at a locus- and gene-specific level. We screened for differential DNA methylation changes in colorectal cancer cells after IR exposure with 2 and 5 Gy. We identified global DNA methylation changes and gene-specific hypomethylation at *bona fide* target genes that were induced by IR exposure.

## Results

### IR induces global DNA methylation changes in colon cancer cells

We previously demonstrated that pretreatment with the DNA methyltransferase inhibitor 5-aza-dC sensitizes colon cancer cells to IR. More specifically, we showed that the level of apoptosis is greater when the cells are treated with a combination of 5-aza-dC and IR than when they are treated with either agent alone, suggesting that the combination of 5-aza-dC and IR is a potential strategy for the treatment of cancer [7]. These data led us to explore whether IR exposure induces DNA methylation changes at both the genome-wide level and the locus- or gene-specific levels in colon cancer.

To determine the DNA methylation changes that occur after IR exposure in colon cancer, we chose HCT116 colorectal cancer cells as a model, because we had shown in a previous study that 5-aza-dC treatment enhances the sensitivity of HCT116 cells to IR. In this study, HCT116 cells were exposed to 2 different IR doses, 2 or 5 Gy, which are known to not induce a high rate of apoptosis with a single treatment. We also treated HCT116 cells with 5-aza-dC (0.5 μM), which served as an internal control for estimating the global demethylation levels.

**Figure 1:**
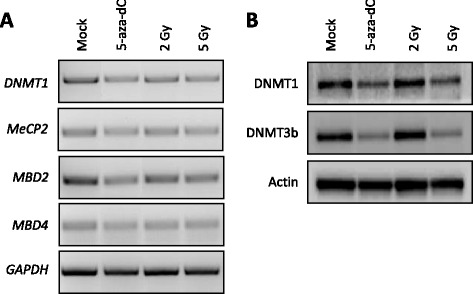
**Ionizing radiation alters the levels of epigenetic regulators in HCT116 cells. (A)** RT-PCR was performed to show the mRNA levels of methylation regulatory factors (*DNMT1*, *MBD2*, *MBD4, and MeCP2*) in HCT116 cells irradiated with 2 or 5 Gy, or treated with 5-aza-dC as a control. **(B)** Western blots show the protein expression levels of DNMT1 and DNMT3b in HCT116 cells irradiated with 2 or 5 Gy, or treated with 5-aza-dC as a control. The blots were probed using anti-DNMT1 (upper panel) and anti-DNMT3b (middle panel) antibodies. *GAPDH* and anti-β-actin were used for normalization for RT-PCR and western blotting analyses, respectively.

First, we determined whether IR treatment of HCT116 cells affected the expression of well-known regulators of DNA methylation, such as DNA-methyltransferase 1 (*DNMT1*), methyl-CpG binding domain proteins 2 and 4 (*MBD2* and *MBD4*), and methyl CpG binding protein 2 (*MeCP2*). Reverse transcriptase polymerase chain reaction (RT-PCR) analyses revealed that these factors were only slightly altered by radiation treatment at the mRNA level (Figure 1A). However, western blot analyses showed that the protein levels of DNMT1 and 3b were decreased in cells exposed to IR (5 Gy) or 5-aza-dC, suggesting that there could be global DNA methylation changes caused by IR treatment (Figure 1B).

To determine genome-wide DNA methylation changes, we performed DNA methylation profiling. HCT116 cells were irradiated at 2 or 5 Gy, or treated with 5-aza-dC for 72 hrs. Next, the isolated DNA was subjected to genome-wide level DNA methylation profiling using the Illumina HumanMethylation450K BeadChip. The irradiated and 5-aza-dC-treated samples were compared to mock-treated samples to determine regions of differential DNA methylation.

**Figure 2:**
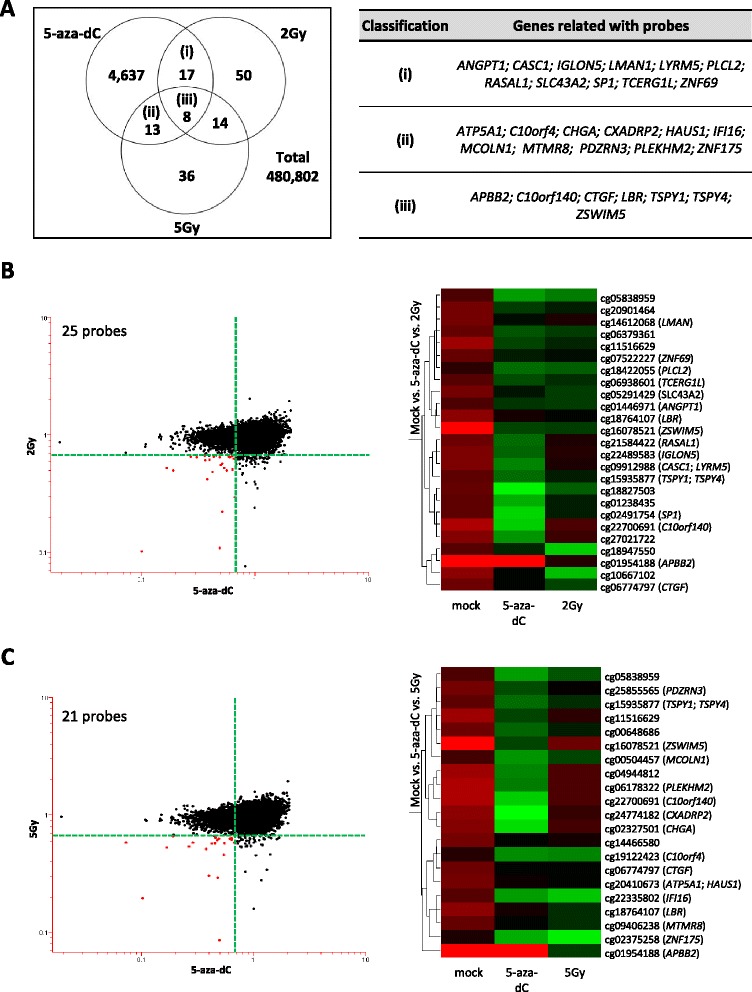
**Identification of radiation-induced hypomethylated candidate genes. (A)** The Venn diagram shows the number of genes displaying differential DNA methylation between 5-aza-dC-treated cells and irradiated cells (2 and 5 Gy). The number of common differentially methylated genes is shown in (i), (ii), and (iii). These common genes were 1.5-fold down-methylated in both 5-aza-dC-treated cells and irradiated cells (2 or 5 Gy). The table shows a summary of candidate genes, which are indicated as (i), (ii), and (iii) **(B, C)** The DNA methylation changes for the indicated cells treated with 5-aza-dC (x-axis) or IR (2 or 5 Gy; y-axis) are plotted by log-fold change, and individual probes are shown in black. Red dots indicate candidate probes that are down-methylated by both 5-aza-dC and IR (2 or 5 Gy) (left panels). Heatmap analysis shows the final candidate genes indicated by red dots in the scatter plots (right panels) and displays the decreasing methylation levels of the 5-aza-dC- and IR-treated groups compared with controls (mock). The color gradient from green to red displays the β-value and ranges from 0 (unmethylated) to 1 (methylated).

The probe call rate was >99% for all of the samples and 454,215 CpG sites out of 485,577 were included in the analysis. To identify hypermethylated loci, we looked for probes or genes that had Δβ-values > I1.5I fold for IR- or 5-aza-dC-treated cells vs. control cells. Interestingly, there were no probes or genes showing hypermethylation in irradiated cells. To identify hypomethylated loci, we set the Δβ-values <1.5 fold for IR- or 5-aza-dC-treated cells vs. control cells. We observed that several gene sets were hypomethylated by IR at similar levels to those observed in 5-aza-dC-treated cells (Figure 2). These data validated reports from previous studies that showed that radiation is linked to global hypomethylation in the genome [8,14,15].

### Identification of radiation-induced hypomethylation in colon cancer cells

Next, we focused on the identification of radiation-induced hypomethylated genes or loci in HCT116 cells. We applied a strict filter to identify the hypomethylated candidate genes induced by radiation at 2 or 5 Gy, or by 5-aza-dC treatment (Δβ-values <1.5 fold compared with mock-treated cells) (Additional file 1: Figure S1). After we excluded non-reliable probes based on Δβ-values in control (mock treated) dataset, we normalized the DNA methylation data by comparing the mock-treated cells with the 5-aza-dC- or IR-treated cells (Additional file 1: Figure S1). In particular, 5-aza-dC, a strong inducer of DNA de-methylation (global hypomethylation), is, an analogue of cytosine, that when incorporated into DNA, irreversibly binds the methyltransferase enzymes as they attempt to methylate the cytosine analogue. This depletion of methyltransferase in the cell results in passive de-methylation, which is known to reactivate epigenetically silenced genes [16]. Therefore, the dataset from the 5-aza-dC-treated cells was a necessary control to help identify the candidate genes that were hypomethylated by radiation. With our criteria (Δβ-values < I1.5I fold), we detected 4,675, 89, and 71 probes in 5-aza-dC-treated cells, 2 Gy-irradiated cells, and 5 Gy-irradiated cells, respectively (Figure 2A). Comparing with irradiated cells and 5-aza-dC, indeed, 25 probes were identified from the 2 Gy-irradiated dataset, and 21 probes were identified from the 5 Gy-irradiated dataset, and probes related 29 candiate genes were identified by comparing with the 5-aza-dC-treated dataset (Figure 2A and Additional file 1: Figure S1). Using heatmap analysis of these probes, we observed the differences in the DNA methylation levels between control cells and 5-aza-dC- or IR-treated cells (Figure 2B, C). We believe that these final candidate genes are truly hypomethylated by radiation.

### Radiation-induced hypomethylated genes show a corresponding increase in gene expression in colon cancer cells

Next, we examined the actual methylation levels of the 29 radiation-induced hypomethylated candidate genes that were identified from our discovery platform (Table 1). In terms of the Δβ-values retrieved from the methylation profile data, most of these genes showed a 1.5- to 11-fold decrease in DNA methylation in the irradiated cells compared with the control cells (Additional file 1: Figure S2). Using 29 candidate genes, we next move on to validate whether gene expression level by RT-PCR of these genes are correlated with their DNA hypomethylation. We realized that *CXADRP2* is a pseudogene and both *TSPY1* and *TSPY4* gene have very high (94%) sequence similarity so that we could not analyze specific their gene expression, therefore, three genes were excepted from our qRT-PCR analysis.

**Table 1:** **Information of target genes**

**Gene**	**Accession No.**	**Function**
*ANGPT1*	NM_001146.3; NM_001199859.1; NM_139290.1	Angiopoietin 1
*APBB2*	NM_001166050.1; NM_001166051.1; NM_001166052.1; NM_001166053.1; NM_001166054.1; NM_004307.1; NM_173075.4	Amyloid Beta (A4) Precursor Protein-Binding, Family B, Member 2
*ATP5A1*	NM_001001935.2; NM_001001937.1; NM_001257334.1; NM_001257335.1; NM_004046.5	ATP Synthase, H+ Transporting, Mitochondrial F1 Complex, Alpha Subunit 1, Cardiac Muscle
*C10orf140*	NM_207371.3	SKI/DACH Domain Containing 1
*C10orf4*	NM_145246.4; NM_203438.2; NM_203439.2; NM_203440.2; NM_203441.2	Fragile Site, Folic Acid Type, Rare, Fra(10)(Q23.3) Or Fra(10)(Q24.2) Candidate 1
*CASC1*	NM_001082972.1; NM_001082973.1; NM_001204101.1; NM_001204102.1; NM_018272.3	Cancer Susceptibility Candidate 1
*CHGA*	NM_001275.3	Chromogranin A (Parathyroid Secretory Protein 1)
*CTGF*	NM_001901.2	Connective Tissue Growth Factor
*CXADRP2*	NR_024387	Coxsackie Virus And Adenovirus Receptor Pseudogene 2
*HAUS1*	NM_138443.3	HAUS Augmin-Like Complex, Subunit 1
*IFI16*	NM_001206567.1; NM_005531.2	Interferon, Gamma-Inducible Protein 16
*IGLON5*	NM_001101372.1	IgLON Family Member 5
*LBR*	NM_002296.3; NM_194442.2	Lamin B Receptor
*LMAN1*	NM_005570.3	Lectin, Mannose-Binding, 1
*LYRM5*	NM_001001660.2	LYR Motif Containing 5
*MCOLN1*	NM_020533.2	Mucolipin 1
*MTMR8*	NM_017677.3	Myotubularin Related Protein 8
*PDZRN3*	NM_015009.1	PDZ Domain Containing Ring Finger 3
*PLCL2*	NM_001144382.1; NM_015184.5	Phospholipase C-Like 2
*PLEKHM2*	NM_015164.2	Pleckstrin Homology Domain Containing, Family M (With RUN Domain) Member 2
*RASAL1*	NM_001193520.1; NM_001193521.1; NM_004658.2	RAS Protein Activator Like 1 (GAP1 Like)
*SLC43A2*	NM_001284498.1; NM_001284499.1; NM_152346.2	Solute Carrier Family 43, Member 2
*SP1*	NM_001251825.1; NM_003109.1; NM_138473.2	Sp1 Transcription Factor
*TCERG1L*	NM_174937.3	Transcription Elongation Regulator 1-Like
*TSPY1*	NM_001197242.1; NM_003308.3	Testis Specific Protein, Y-Linked 1
*TSPY4*	NM_001164471.1	Testis Specific Protein, Y-Linked 4
*ZNF175*	NM_007147.2	Zinc Finger Protein 175
*ZNF69*	NM_021915.2	Zinc Finger Protein 69
*ZSWIM5*	NM_020883.1	Zinc Finger, SWIM-Type Containing 5

**Figure 3:**
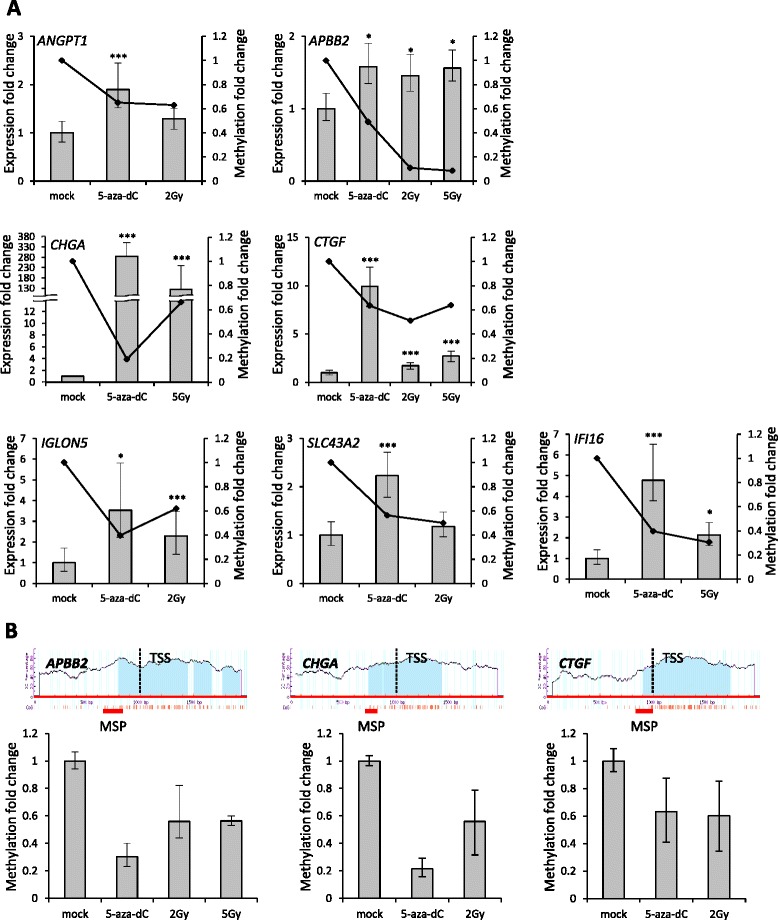
**Correlation between DNA hypomethylation and gene expression of candidate genes. (A)** Seven genes (*ANGPT1*, *APBB2*, *CHGA*, *CTGF*, *IFI16*, *IGLON5*, and *SLC43A2*) showed a correlation between their levels of DNA hypomethylation and gene expression. The methylation fold-change values were obtained from the HumanMethylation450K profile, and the gene expression fold-change values were quantified by qRT-PCR from HCT116 cells with either 5-aza-dC treatment (0.5 μM) or IR (2 or 5 Gy). The bar graphs indicate the gene expression level, and the line charts indicate the methylation level. **(B)** Quantitative MSP (qMSP) analysis of the CpG island of the *APBB2*, *CHGA*, and *CTGF* gene are shown. The promoter region (defined as upstream and downstream 1 kb from the transcriptional start site; TSS) includes typical CpG islands. The MSP primer location is indicated with a red bar. Each bar graph indicates the DNA methylation level of the *APBB2*, *CHGA*, and *CTGF* gene by qMSP, respectively. The candidate gene methylation values were normalized to the methylation of an *Alu* element. *P < 0.05; ***P < 0.001.

It is well known that DNA methylation changes can alter the transcriptional expression level of genes. To determine if the aberrant DNA methylation observed in the irradiated and 5-aza-dC-treated cells caused transcriptional gene expression changes, we measured the mRNA expression level of these genes by quantitative RT-PCR (qRT-PCR) analysis. We plotted the gene expression changes with the methylation changes to determine whether the level of demethylation or hypomethylation at each gene corresponded with the level of increased gene expression. Importantly, seven genes (*ANGPT1, APBB2, CHGA, CTGF, IFI6, IGLON5*, and *SLC43A2*) showed the expected correlation between demethylation or hypomethylation by 5-aza-dC or radiation treatments, and increased mRNA expression levels (Figure 3A). Therefore, we identified seven radiation-induced hypomethylated genes with increased gene expression in colon cancer cells.

To verify the methylation status of these genes, we used methylation-specific PCR (MSP) analysis. We tested three (*APBB2, CHGA, and CTGF)* genes out of the seven genes as a representative example. As shown in Figure 3B, we confirmed the methylation status of the CpG islands around the *APBB2* , *CHGA, and CTGF* by quantitative methylation-specific PCR (qMSP). These data confirmed that the *these genes* are strong candidate hypomethylated genes by IR.

### Radiation-induced hypomethylated genes show decreased levels of DNMT1 in their promoter regions

**Figure 4:**
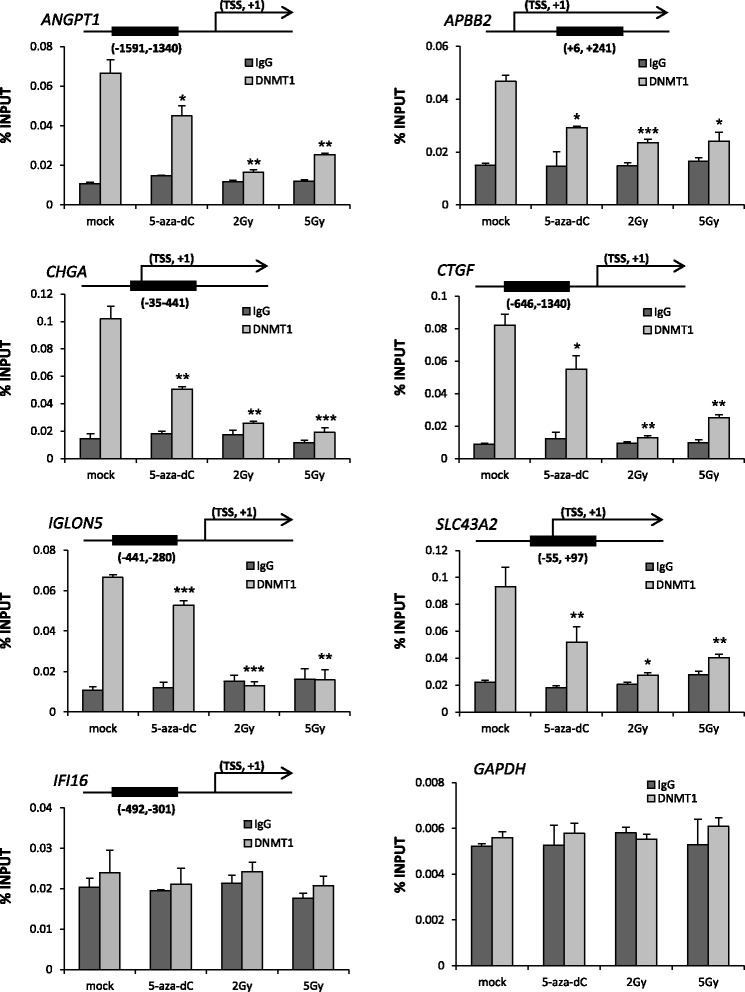
**The interactions of DNMT1 with the promoter regions of candidate genes following IR treatment.** Chromatin immunoprecipitation (ChIP) assays were performed to determine the levels of DNMT1 at the promoter regions of candidate genes (*ANGPT1*, *APBB2*, *CHGA*, *CTGF*, *IFI16*, *IGLON5*, and *SLC43A2*) in HCT116 cells following 0.5 μM 5-aza-dC treatment or irradiation (2 or 5 Gy). *GAPDH* was used as negative control. Cross-linked and sheared chromatin was immunoprecipitated with an anti-IgG antibody (dark gray bar) or with anti-DNMT1 (light gray bar). The results are shown as a percentage of the input chromatin. Associated chromatin in the ChIP samples were quantified by real-time RT-PCR. The data represents the mean ± the standard deviation of triplicates. Representative data are shown from three independent trials. *P < 0.05; **P < 0.01; ***P < 0.001; TSS, Transcription Start Site.

As shown in Figure 1, DNMT1 and 3b are downregulated in irradiated cells. DNMTs are enzymes that are involved in a critical step of the DNA methylation process [17]. In cancer cells, DNMT1 is responsible for the majority of the DNA-methylation capacity [18,19], and it is a contributing factor in maintaining the abnormal promoter methylation in tumor cells. Therefore, we asked whether these genes might be directly associated with DNMT1 protein in terms of occurring demethylation after irradiation. Using chromatin immunoprecipitation (ChIP) analysis, we examined the correlation between DNMT1 and the CpG island located in the promoter region of each candidate gene in HCT116 cells (Figure 4). We observed that DNMT1 levels were decreased in the promoter regions of most of the candidate genes (*ANGPT1, APBB2, CHGA, CTGF, IFI6, IGLON5*, and *SLC43A2*). Among the candidate genes, DNMT1 levels were decreased from 21 to 50% by 5-aza-dC treatment, from 50 to 81% by 2 Gy IR treatment, and from 48 to 81% by 5 Gy IR treatment compared with controls. For the *IFI16* gene, we observed there is no correlation between DNMT1 level of its promoter region and its gene expression level along with DNA hypomethylation level. These data strongly support a correlation between decreased DNMT1 levels and DNA hypomethylation in the promoter regions of the candidate genes with elevated gene expression (Figure 4). Taken together, our validation strategy solidifies the potential biological relevance of these newly identified genes that are hypomethylated by radiation in colon cancer cells. These findings suggest novel epigenetic roles for these previously uncharacterized genes in colon cancer.

Although we identified a handful of genes that were hypomethylated following irradiation, we wanted to determine whether these genes are associated with a particular cellular pathway or network. We analyzed our candidate gene list using well-curated databases (GO, GeneMANIA and KEGG) that include a variety of biological processes, molecular functions, and cellular components (Additional file 1: Figure S3). The possible roles of the candidate genes can be hypothesized by looking at the various signaling pathways predicted by KEGG pathway analysis (Additional file 2: Table S2). Collectively, these data suggested that the radiation-induced hypomethylated genes play important roles not only in response to radiation, but also in cellular regulatory events.

## Discussion

DNA methylation plays a crucial role in the regulation of gene expression and chromatin organization within normal eukaryotic cells. In cancer, global patterns of DNA methylation are altered. Tumor cells often show global hypomethylation of repeat elements and hypermethylation of promoter regions of tumor suppressor genes or a subset of CpG-dense gene-associated regions (CpG islands).

We previously showed that 5-aza-dC pretreatment increases the radiosensitivity of colon cancer cells, and that the combination of 5-aza-dC treatment and IR promotes apoptosis, suggesting that this combination treatment could be useful for clinical applications [7]. Based on this previous work, we proposed to examine the DNA methylation level of each gene or loci in the genome that could potentially be involved in radiosensitivity. This information is also important for determining the proper use of demethylation agents as radiotherapy sensitizers or chemotherapeutics. Therefore, here, we performed genome-wide DNA methylation analysis of colon cancer cells after treatment with 5-aza-dC or IR to examine the DNA methylation alterations, and we showed that IR treatment of cancer cells resulted in extensive changes in the DNA methylation status of genes.

Other possible mechanism for radiation-induced global demethylation is the relocalization of DNMTs from the nucleus to the cytoplasm [20], which may cause a change in the DNA methylation maintenance machinery in the genome. In addition, it is possible that radiation promotes the enzymatic replacement of 5-methylcytosine with cytosine [21].

Although the mechanisms underlying this radiosensitization effect are not well understood, several studies have shown that radiation induces global hypomethylation in human cancers, as evidenced by the depleted expression levels of DNMT1, MBP proteins, and MeCP2 [22-24]. Here, we have focused on identification of radiation induced hypomethylated genes in colon cancer cells. Using genome-wide DNA methylation array, we have demonstrated the radiation effect on global genome or specific gene as a model system to provide evidence for radiation induced hypomethylation.

Previously, several tumor- and proliferation-associated genes were found to be hypomethylated in human cancers. For example, the *pS2* gene, which encodes a pleotropic factor implicated in the control of cell proliferation, shows cancer-associated hypomethylation and the concordant changes in gene expression [25]. In addition, radiation induces hypomethylation and expression of urinary-type plasminogen activator (*uPA*) in meningioma, which could contribute to meningioma progression *in vitro* and *in vivo* [22]. However, there is still little information on radiation-induced hypomethylation of specific genes in cancer. In the present study, we showed radiation-induced hypomethylation in colon cancer cells, and we identified several hypomethylated genes that also showed upregulated gene expression and downregulated DNMT1 levels in their promoter regions.

It has been known that radiation has been shown to induce global hypomethylation [20]. In terms of losing DNA methylation level at globally, decreased genomic DNA methylation was observed at 24–72 hrs post-irradiation in cell lines and this could be due to decreased levels of DNA methyltrasferases (DNMTs). Although several previous reports have done to see the genomic hypomethylation with irradiation after different time points and showed global hypomethylation with different profiles without specific loci or genes [26], we limited one time point (72 hrs after 2Gy and 5Gy irradiation) and interestingly we have indeed reached to identification of purely IR induced hypomethylated genes along with global hypomethylation at the genome level.

To date, DNA methylation profiling to determine responses to ionizing radiation has not been well described. Kim et al. performed DNA methylation profiling to compare a radioresistant lung cancer cell line to a radiosensitive lung cancer cell line using Illumina BeadChip to query 27,578 CpG sites [27]. They found 1,091 genes that were differentially methylated. However, they did not show that the differentially methylated genes were functionally associated with the radiosensitive phenotype. More recently, Antwih et al. identified DNA methylation changes induced by IR using the HumanMethylation450K array platform in breast cancer cell line [24]. They suggested that that the differentially methylated genes were associated with cellular processes, such as cell cycle, DNA repair, and apoptosis pathways. Although similar approach applied to this study with our work, this study differs from our work in terms of identification of radiation induced hypomethylated genes. In our strick criteria, we did not see any genes are hypermetylated by IR, therefore we could focused on radiation induced hypoemthyaltion and the fact is that actual our candidate genes are hypomethylated (demethylated) in not only in irradiated cells but also 5-aza-dC treated cells as well as downregulated their gene expression by RT-PCR in both, which Specfically, while this work demonstrated that rdadiation may induce DNA methylation changes and identify the loci-specific changeds in DNA methylation as we do here, the expereimetnal for validation for target genes was not designed to show if these gene expression are regulated by DNA methylation by IR. In our study, genome-wide DNA methylation array was useful to identify new genes are induced hypemethylation of them by IR and we can stress here that these genes were newly identified genes are hypomethylated by IR with specific validation by several experimental tools such as qRT-PCR to confirm the correlation between DNA methylation and gene expression and to see if these genes are actually targeted by DNMT at the transcriptional level by ChIP assaye transcriptional level. Since this study did not use 5-aza-dC treated cells.

IR exposure results in the simultaneous activation or downregulation of multiple signaling pathways that play critical roles in cell type-specific control of survival or death. Although IR is a well-known genotoxic agent and human carcinogen [6], very recent studies have suggested that epigenetic mechanisms that are associated with gene silencing, such as histone modifications and DNA methylation, may be involved in regulating radiosensitivity in cancer cells. A number of previous studies have reported that several histone deacetylase inhibitors are cytotoxic and can sensitize tumor cells to radiotherapy. However, little information is available regarding the effects of DNMT inhibitors on radiosensitization [12,28]. Our previous work demonstrated that global DNA methylation changes, especially demethylation by 5-aza-dC, could be important for radiosensitization. We showed that the combination of 5-aza-dC and IR treatment results in the apoptosis of colon cancer cells, suggesting that this combination could be a novel cancer treatment. In this study, we further demonstrated that radiation induces hypomethylation of site-specific genes in colon cancer cells.

We searched the literature to identify the biological functions of our *bona fide* radiation-induced hypomethylated genes. The amyloid beta A4 precursor protein-binding, family B, member 2 (*APBB2*) gene encodes a protein that binds to amyloid beta precursor protein (APP), which is central to the pathogenesis of Alzheimer’s disease [29]. In addition, overexpression of *APBB2* in PC12 cells inhibits cell cycle progression through its nuclear translocation and inhibition of thymidilate synthase, which ultimately leads to a cell cycle delay [30]. Its role in cell cycle regulation could also be related to the role of *APBB2* in neurodegeneration. Chromogranin A (*CHGA*) regulates catecholamine storage and release through intracellular and extracellular mechanisms [31]. *CHGA* is also a candidate gene that contributes to autonomic dysfunction syndromes, including the intermediate phenotypes that contribute to hypertension [31]. *Chga*-null mice show abnormal changes in the autonomic control of circulation, further suggesting a role for *CHGA* in hypertension risk [32]. Connective tissue growth factor (*CTGF*) is also known as insulin-like growth factor-binding protein 8 (*IGFBP8*). There is little information regarding the biological function of the *CTGF* gene in cancer. However, a polymorphism in the promoter of the *CTGF* gene, accompanied by its transcriptional repression, is associated with susceptibility to systemic sclerosis [33]. In addition, Nakanish et al. showed that the *CTGF* gene may be involved in bone and testis development using transgenic *Ctgf*-overexpressing mice [34]. L-type amino acid transporter 4 (*LAT4*, also known as *SLC43A2*) mediates the sodium-independent transport of bulky neutral amino acids across cell membranes [35]. Taken together, we have identified genes of unknown function in radiosensitivity that are hypomethylated in response to radiation and show corresponding alterations in DNMT1 association and gene expression in colon cancer cells.

## Conclusion

In summary, we have analyzed the genome--wide CpG methylation profiles of HCT116 cells to identify IR induced DNA methylation changes. We found that IR induces DNA hypomethylation, rather than hypermethylation, at the genome-wide level by comparing with the DNA methylation profiles of 5-aza-dC treated cells. Among these DNA methylation changes, we found *bona fide* site-specific genes that were hypomethylated by IR. Furthermore, these genes showed increased gene expression and decreased levels of DNMT1 in their promoter regions that correlated with their degree of hypomethylation. Therefore, these data strongly suggest that radiation exposure plays a critical role in regulating epigenetic alterations in human cancer.

## Methods

### Cell culture and 5-Aza-dC treatment

The human colorectal carcinoma cell line HCT116 was obtained from ATCC and cultured in McCoy’s 5A medium (WelGENE) with 10% fetal bovine serum (Hyclone) and 1% penicillin/streptomycin (Gibco) at 37°C in a humid incubator with 5% CO_2_. The cells were treated with 0.5 μM 5-aza-dC (Sigma) for 3 days, replacing media and 5-aza-dC every 24 hrs.

### Irradiation

The cells were exposed to gamma rays through the air-center mode after changing to fresh media. All irradiations were performed using a ^137^Cs-ray source (Eckert & Ziegler) at a dose rate of 2.6 Gy/min. Following irradiation at doses of 2 and 5 Gy, the cells were incubated for 3 days at 37°C in a humid incubator with 5% CO_2_.

### Methylation array

The Infinium HumanMethylation450K BeadChip (Illumina) includes 485,577 CpG sites located throughout the genome [36]. Genomic DNA from HCT116 cells [mock, irradiated (2 and 5 Gy), and 5-aza-dC (0.5 μM for 72 hrs) treated] was isolated using a phenol/chloroform method. Bisulfite modification of genomic DNA was performed using an EZ DNA Methylation Kit (Zymo Research). The bisulfite conversion efficiency was determined by sample-dependent controls on the chip and was displayed in the quality control panel in the software. All samples passed quality control measurements. The samples were run on an Infinium HumanMethylation450K BeadChip (Illumina) and scanned on an Illumina iScan instrument according to the manufacturer’s instructions. The methylation values for individual CpG sites in each sample were obtained as β-values. The β-value generated for each CpG locus measure the intensity of methylated (β = 1) and unmethylated probes (β = 0). The β-value is a continuous variable that is calculated by dividing the intensity of the methylated beads by the combined intensity, and these values range from 0 to 1. A CpG locus was considered differentially methylated if the β-value was > = |0.2| and its ratio was > |1.5|X fold compared to control (mock treated). This cut-off value of |0.2| represents the 99% confidence interval of the detection limit [13].

### Hierarchical clustering

>I1.5I fold demethylated (hypomethylated) probes were independently analyzed in control (mock treated) vs. 5-aza-dC treated cells, control vs. 2 Gy irradiated cells or control vs. 5 Gy irradiated cells. The selected probes were 4,675, 89 and 71 probes, respectively. Commonly hypoemthylaed probes were collected between control vs. 5-aza-dC treated cells and control vs. 2 Gy irradiated cells, or between and control vs. 5 Gy irradiated cells via Venn diagram analysis, and finally common hypomethylated probes (25 and 21 probes, respectively) were analyzed. Hierarchical clustering was performed with Agilent GeneSpring GX 7.3 software using common hypomethylated probes. Pearson Correlation was used for similarity measurement.

### Western blotting

Western blotting was performed as described previously [37]. The antibodies used for western blot analysis were anti-DNMT1 (Sigma), anti-DNMT3a (Sigma), and anti-β-actin (Sigma).

### cDNA synthesis and quantitative RT-PCR

The cells were trypsinized and homogenized using a QIAshredder Kit (QIAGEN). Total RNA from the cells was extracted using RNeasy Mini Kit (QIAGEN). The RNA quantity was measured using a NanoDrop 2000/2000c instrument (Thermo Scientific). For cDNA synthesis, 1 μg of total mRNA was reverse-transcribed into cDNA using the iScript^TM^ cDNA Synthesis Kit (BioRad). The reaction was performed at 25°C for 5 min and then 42°C for 30 min, and was terminated by heating to 85°C for 5 min.

The target gene transcripts were analyzed by qRT-PCR, which was performed using a CFX96^TM^ Real-Time PCR Detection System (BioRad). All primers were designed using the Primer3 program (http://bioinfo.ut.ee/primer3/) and are listed in the Additional file 2: Table S1. For normalization, human *GAPDH* was amplified.

### Bisulfite modification and quantitative methylation-specific PCR (qMSP)

Genomic DNA was isolated from HCT116 cells using a phenol/chloroform method. Bisulfite modification of 2 μg of genomic DNA was performed using an EZ DNA Methylation Kit (Zymo Research). For positive and negative controls, *in vitro* methylated DNA (IVD) and H_2_O were treated, respectively. For methylation analysis of target genes, qMSP amplification was performed on bisulfite-treated samples and normalized by an *Alu* element. All primers were designed using MethPrimer (http://www.urogene.org/cgi-bin/methprimer/methprimer.cgi) and are presented in Additional file 2: Table S1. qRT-PCR was performed with a CFX96^TM^ Real-Time PCR Detection System (BioRad).

### Chromatin immunoprecipitation (ChIP)

ChIP assays were performed as previously described [38]. PCR was performed using a C1000 Thermal Cycler (BioRad) with the ChIP primers that are listed in Additional file 2: Table S1. An anti-DNMT1 antibody (Sigma) was used to immunoprecipitate the DNMT1-associated chromatin fragments.

### Ethics statement

Human colorectal carcinoma cell line HCT116 used in this study is publically available through ATCC.

## Additional files

Additional file 1: Figure S1. Overview of the screening approach using the HumanMethylation450K platform that was performed in this study. **Figure S2.** Screen validation by assessing the correlation between gene expression and methylation levels of the candidate genes identified from the HumanMethylation450K platform. We assessed the gene expression level of the 26 hypomethylated genes that were identified from the methylation using RT-PCR to determine if there was a correlation between their gene expression and methylation levels. The bar graphs indicate their gene expression level and the line charts indicate their methylation level. **Figure S3.** Gene network analysis of candidate genes visualized by Cytoscape. The gene interaction analysis, excluding *CXADRP2* (pseudogene), was performed using GeneMANIA and visualized by the Cytoscape 3.0.2 program. The black circles indicate candidate genes and the gray circles represent interacting genes. *CASC1* and *LYRM5* failed to show any interactions. Of the gene interactions, there were six types of evidence codes, each indicated with a different color node: co-expression (40.92%), physical interactions (35.98%), pathway (12.29%) co-localization (7.3%), shared protein domains (1.79%), and genetic interactions (1.73%). Most of the genes were intimately connected and showed strong interrelationships. The predicted cellular functions, GO terms, and related genes for each category, are presented in the table (lower panel). These functional predictions are statistically significant, and the q-value of the categories range from 8.02 × 10^−2^ to 3.10 × 10^−6^. Additional file 2: Table S1. Primer information for experiments. **Table S2.** Signaling pathway of target genes by KEGG database.
