# Supplementary material for: Identification of radiation-induced aberrant hypomethylation in colon cancer
Source: BMC Genomics. 2015 Feb 6;16(1):56. doi: 10.1186/s12864-015-1229-6 (PMC4342812; doi:10.1186/s12864-015-1229-6)
Supplement: Additional file 1: Figure S1. — Overview of the screening approach using the HumanMethylation450K platform that was performed in this study. Figure S2. Screen validation by assessing the correlation between gene expression and methylation levels of the candidate genes identified from the HumanMethylation450K platform. We assessed the gene expression level of the 26 hypomethylated genes that were identified from the methylation using RT-PCR to determine if there was a correlation between their gene expression and methylation levels. The bar graphs indicate their gene expression level and the line charts indicate their methylation level. Figure S3. Gene network analysis of candidate genes visualized by Cytoscape. The gene interaction analysis, excluding CXADRP2 (pseudogene), was performed using GeneMANIA and visualized by the Cytoscape 3.0.2 program. The black circles indicate candidate genes and the gray circles represent interacting genes. CASC1 and LYRM5 failed to show any interactions. Of the gene interactions, there were six types of evidence codes, each indicated with a different color node: co-expression (40.92%), physical interactions (35.98%), pathway (12.29%) co-localization (7.3%), shared protein domains (1.79%), and genetic interactions (1.73%). Most of the genes were intimately connected and showed strong interrelationships. The predicted cellular functions, GO terms, and related genes for each category, are presented in the table (lower panel). These functional predictions are statistically significant, and the q-value of the categories range from 8.02 × 10^−2^ to 3.10 × 10^−6^. [file 12864_2015_1229_MOESM1_ESM.ppt]

## Slide 1
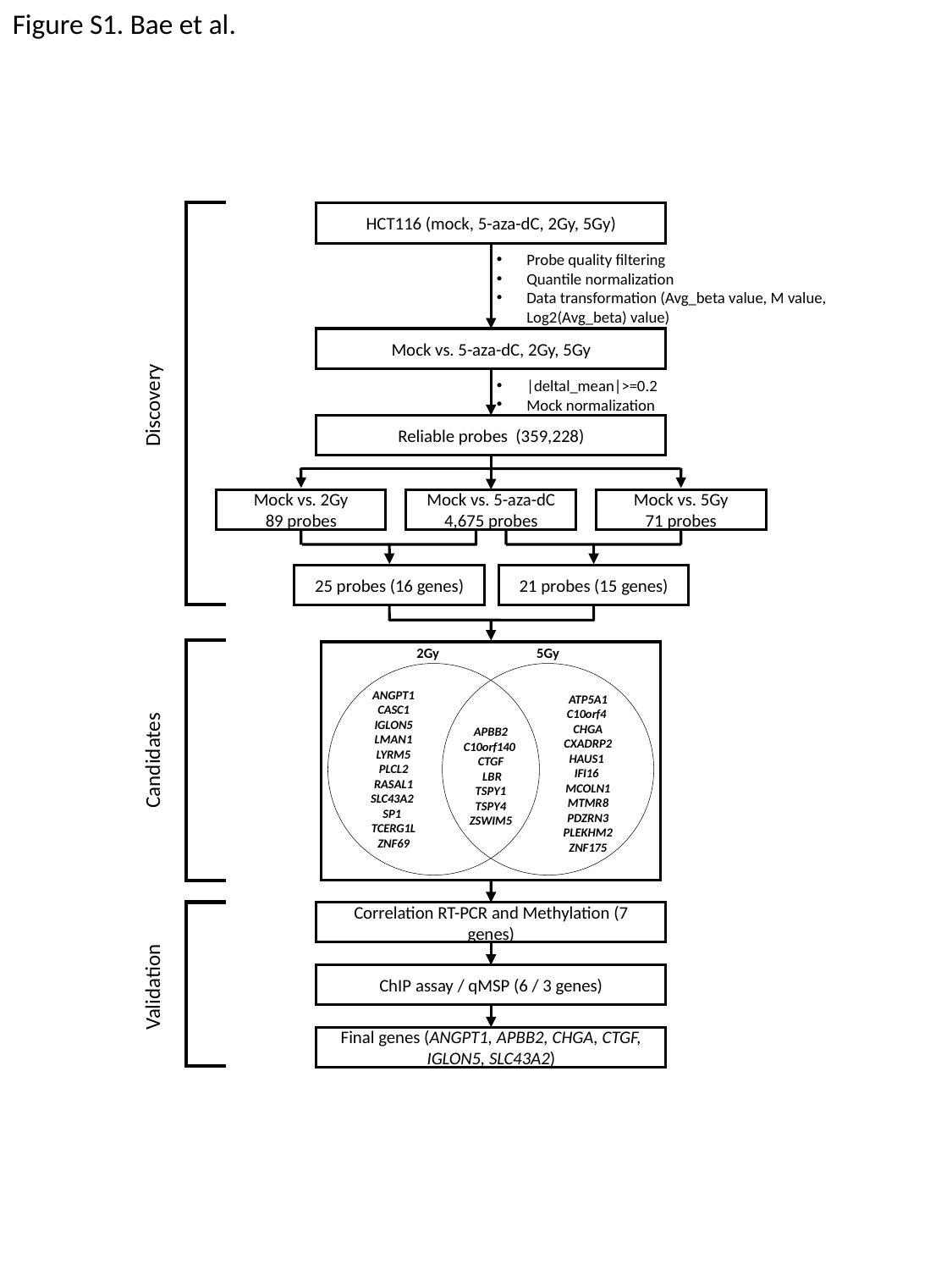

Figure S1. Bae et al.
HCT116 (mock, 5-aza-dC, 2Gy, 5Gy)
Probe quality filtering
Quantile normalization
Data transformation (Avg_beta value, M value, Log2(Avg_beta) value)
Mock vs. 5-aza-dC, 2Gy, 5Gy
|deltal_mean|>=0.2
Mock normalization
Discovery
Reliable probes (359,228)
Mock vs. 2Gy
89 probes
Mock vs. 5-aza-dC
4,675 probes
Mock vs. 5Gy
71 probes
25 probes (16 genes)
21 probes (15 genes)
2Gy
5Gy
ANGPT1 CASC1
IGLON5 LMAN1
LYRM5
PLCL2
RASAL1 SLC43A2
SP1
TCERG1L ZNF69
ATP5A1 C10orf4
CHGA
CXADRP2
HAUS1
IFI16
MCOLN1 MTMR8 PDZRN3
PLEKHM2 ZNF175
APBB2
C10orf140
CTGF
 LBR
TSPY1
TSPY4
ZSWIM5
Candidates
Correlation RT-PCR and Methylation (7 genes)
ChIP assay / qMSP (6 / 3 genes)
Validation
Final genes (ANGPT1, APBB2, CHGA, CTGF, IGLON5, SLC43A2)

## Slide 2
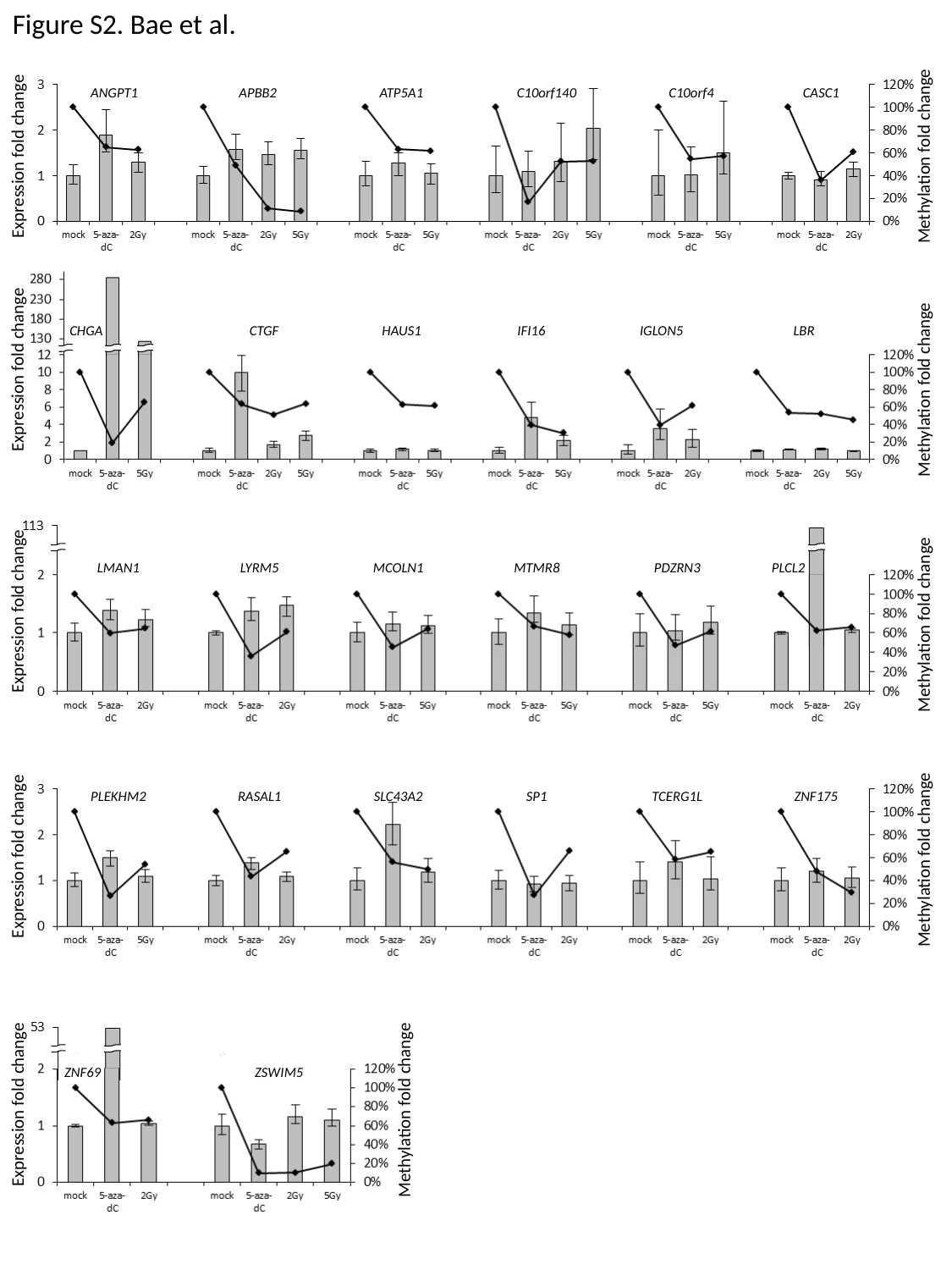

Figure S2. Bae et al.
ANGPT1
APBB2
ATP5A1
C10orf140
C10orf4
CASC1
Expression fold change
Methylation fold change
CHGA
CTGF
HAUS1
IFI16
IGLON5
LBR
Expression fold change
Methylation fold change
LMAN1
LYRM5
MCOLN1
MTMR8
PDZRN3
PLCL2
Expression fold change
Methylation fold change
PLEKHM2
RASAL1
SLC43A2
SP1
TCERG1L
ZNF175
Expression fold change
Methylation fold change
ZNF69
ZSWIM5
Expression fold change
Methylation fold change

## Slide 3
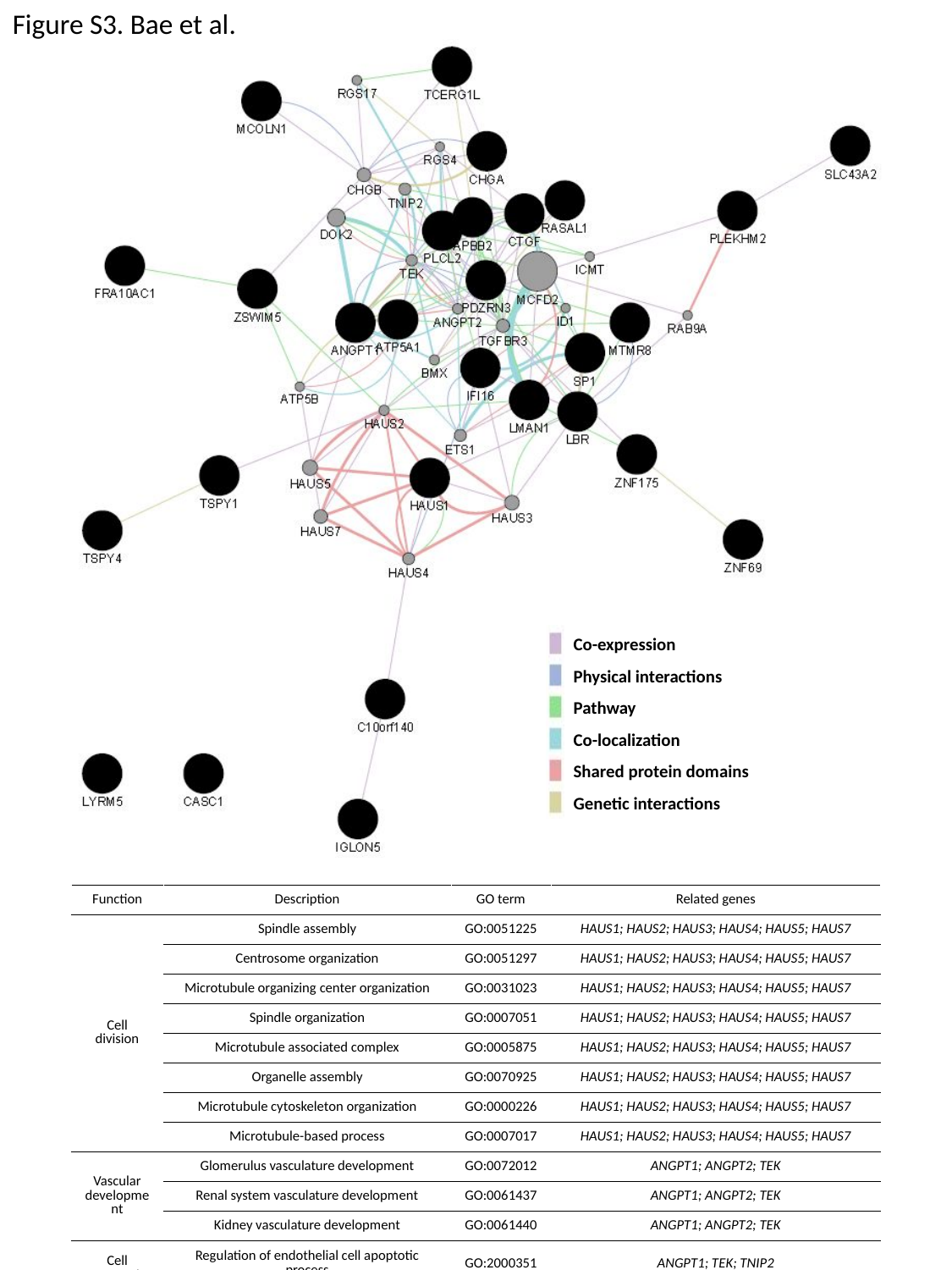

Figure S3. Bae et al.
Co-expression
Physical interactions
Pathway
Co-localization
Shared protein domains
Genetic interactions
| Function | Description | GO term | Related genes |
| --- | --- | --- | --- |
| Cell division | Spindle assembly | GO:0051225 | HAUS1; HAUS2; HAUS3; HAUS4; HAUS5; HAUS7 |
| | Centrosome organization | GO:0051297 | HAUS1; HAUS2; HAUS3; HAUS4; HAUS5; HAUS7 |
| | Microtubule organizing center organization | GO:0031023 | HAUS1; HAUS2; HAUS3; HAUS4; HAUS5; HAUS7 |
| | Spindle organization | GO:0007051 | HAUS1; HAUS2; HAUS3; HAUS4; HAUS5; HAUS7 |
| | Microtubule associated complex | GO:0005875 | HAUS1; HAUS2; HAUS3; HAUS4; HAUS5; HAUS7 |
| | Organelle assembly | GO:0070925 | HAUS1; HAUS2; HAUS3; HAUS4; HAUS5; HAUS7 |
| | Microtubule cytoskeleton organization | GO:0000226 | HAUS1; HAUS2; HAUS3; HAUS4; HAUS5; HAUS7 |
| | Microtubule-based process | GO:0007017 | HAUS1; HAUS2; HAUS3; HAUS4; HAUS5; HAUS7 |
| Vascular development | Glomerulus vasculature development | GO:0072012 | ANGPT1; ANGPT2; TEK |
| | Renal system vasculature development | GO:0061437 | ANGPT1; ANGPT2; TEK |
| | Kidney vasculature development | GO:0061440 | ANGPT1; ANGPT2; TEK |
| Cell apoptosis | Regulation of endothelial cell apoptotic process | GO:2000351 | ANGPT1; TEK; TNIP2 |
| | Endothelial cell apoptotic process | GO:0072577 | ANGPT1; TEK; TNIP2 |
| Cell migration | Endothelial cell migration | GO:0043542 | ANGPT1; ANGPT2; TEK; ID1 |

## Slide 4
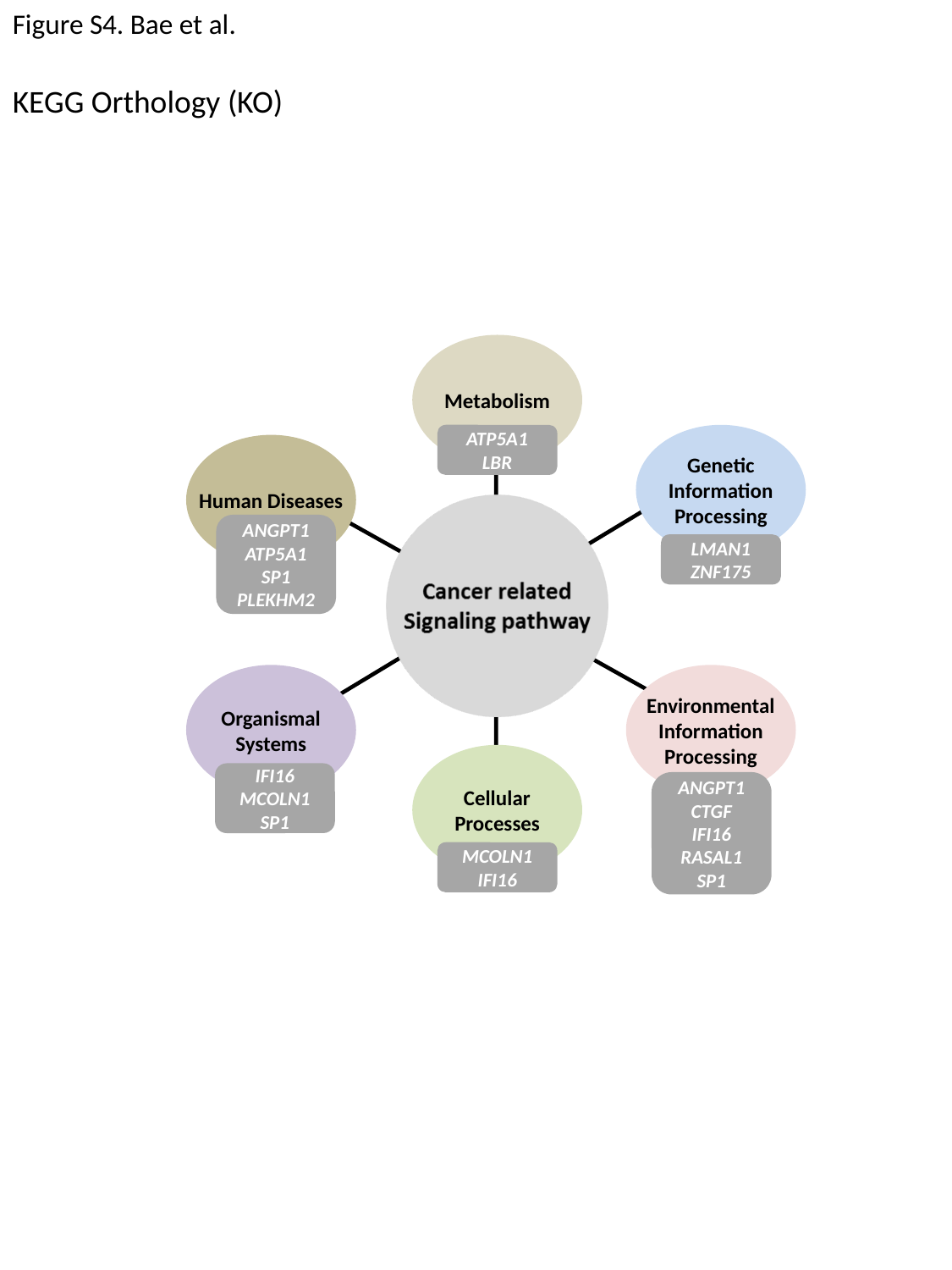

Figure S4. Bae et al.
KEGG Orthology (KO)
Metabolism
ATP5A1
LBR
Genetic Information Processing
Human Diseases
ANGPT1
ATP5A1
SP1
PLEKHM2
LMAN1
ZNF175
Organismal Systems
Environmental Information Processing
Cellular Processes
IFI16
MCOLN1
SP1
ANGPT1
CTGF
IFI16
RASAL1
SP1
MCOLN1
IFI16
