# Supplementary material for: Identification of radiation-induced aberrant hypomethylation in colon cancer
Source: BMC Genomics. 2015 Feb 6;16(1):56. doi: 10.1186/s12864-015-1229-6 (PMC4342812; doi:10.1186/s12864-015-1229-6)
Supplement: Additional file 2: Table S1. — Primer information for experiments. Table S2. Signaling pathway of target genes by KEGG database. [file 12864_2015_1229_MOESM2_ESM.doc]

| **Table S1. Primer information for experiments.** | | | |
| --- | --- | --- | --- |
| **Gene** | **Target Transcript** | **Realtime RT-PCR** | |
|  |  | **Forward Primer (5’-3’)** | **Reverse Primer (5’-3’)** |
| *ANGPT1* | NM_001146.3 | CCGGATTCAACATGGGCAAT | CTGCTGTATCTGGGCCATCT |
| *APBB2* | NM_001166050.1; NM_004307.1; NM_173075.4 | CGATCCTCCCACAATGAACT | AGCTGGTTCTCCCCATTTTT |
| *ATP5A1* | NM_001001937.1 | TTTTGCCCAGTTCGGTTCTG | GATATCCCCTTACACCCGCA |
| *C10orf140* | NM_207371.3 | TAACAACAAGTGGGGGTGGT | GAGCACTGGAGCCAATCACT |
| *C10orf4* | NM_145246.4 | GCCGGGAAAGAAGGAAAC | GGATTCTCCACAGCGTTCAT |
| *CHGA* | NM_001275.3 | TGTCCTGGCTCTTCTGCTCT | TGGAAAGTGTGTCGGAGATG |
| *CTGF* | NM_001901.2 | TACCAATGACAACGCCTCCT | CCGTCGGTACATACTCCACA |
| *HAUS1* | NM_138443.3 | TTTCAGAACGCAACAGGGTC | AGTGCTAGAGAGATTGGCGG |
| *IFI16* | NM_005531.2 | CCACAAGCAGCACTGTCAAA | GTTGGGTGGAGCTGACAATG |
| *IGLON5* | NM_001101372.1 | GTGTGTGAAGGTGACAACGC | CGGTGATGAGGATGGAGAAC |
| *LBR* | NM_002296.3; NM_194442.2 | ATTTCCCTCCTCCTTTGCCA | CTCTACGCCCTGGAAGAGAG |
| *LMAN1* | NM_005570.3 | CCCTGTGTTTGGATCAGCTG | AGGATAGGGTTTGTTGCGGA |
| *LYRM5* | NM_001001660.2 | CCGCCCCCTTTACTGACA | TGGCCATTTTCATTTATCATGT |
| *CASC1* | NM_001082972.1; NM_001082973.1; NM_018272.3 | TCTGCTTTTGCATTTGGTTG | TGCTTCAGAAAATGCCTCAC |
| *MCOLN1* | NM_020533.2 | GAGCGGCTGGAATTTGTCAA | AAGGTCAGGTAGCGGATCAC |
| *MTMR8* | NM_017677.3 | ATCACTAGCCTGGGTTGTCC | TTCCCATACGCCCAAAGTCT |
| *PDZRN3* | NM_015009.1 | GAGGGCTGGATGGATGATGA | GTTGGACAAGATGGTGGCTG |
| *PLCL2* | NM_001144382.1; NM_015184.5 | TAGGCACCAAGGAAGGTTTG | CGGTGCCTGGTTTATTCAGT |
| *PLEKHM2* | NM_015164.2 | AGAACCCCTTCAACGAGGAG | GCCTCCTCATCTGATCTGCT |
| *RASAL1* | NM_004658.2 | ACGTGTGAGAGACTTCCTGG | GACGTAGCGCTTCTTGAAGG |
| *SLC43A2* | NM_152346.2 | AGGAGGTGAGCTGGATGAAC | AATCAGCAAGCAGGAAACCG |
| *SP1* | NM_003109.1; NM_138473.2 | CACCACTCTCACACCCATTG | TCCACCTGCTGTGTCATCAT |
| *TCERG1L* | NM_174937.3 | ACAGGATCATTGAGGACCCG | TCCTTGTGCCTTTGTCCTCT |
| *ZNF175* | NM_007147.2 | GCTTACTGGACGCTGAAGTG | AAGAGAGATCCTGCAGCCTC |
| *ZNF69* | NM_021915.2 | ACGCTATGCCCTGCTGTAGT | GTTTCTCCTGGGGTTTTGGT |
| *ZSWIM5* | NM_020883.1 | GAGAACGTGCTGCAAGTCG | TCATCTGGAAAAGGGTTTCG |
| *DNMT1* | NM_001130823.1 NM_001379.2 | GCACAAACTGACCTGCTTCA | GCCTTTTCACCTCCATCAAA |
| *MeCP2* | NM_004992.3 | ACTCCCCAGAATACACCTTGCTT | TGAGGCCCTGGAGGTCCT |
| *MBD2* | NM_003927.4 | AGTGAAATCAGACCCACAACGAA | CATCTGATGCACTAAGTCCTTGTAGC |
| *MBD4* | NM_001276270.1 NM_001276271.1 NM_001276272.1 NM_001276273.1 NM_003925.2 | TCTAGTGAGCGCCTAGTCCCAG | TTCCAATTCCATAGCAACATCTTCT |
| *GAPDH* | NM_002046.5 | GAAGGTCGGAGTCAACGGATTT | ATGGGTGGAATCATATTGGAAC |
| **Gene** | **locus** | **ChIP assay** | |
|  |  | **Forward Primer (5’-3’)** | **Reverse Primer (5’-3’)** |
| *ANGPT1* | chr8 (108,511,593  -108,511,842) | CACTTGCCAGCTCTGTGAAT | ACCACTTTTGCAGCCTCTTG |
| *APBB2* | chr4 (41,216,396  -41,216,630) | AGCCTGGAGAAGTGGAATCT | AAGGCAAGGAAGAAAGCTGG |
| *CHGA* | chr14 (93,389,410  -93,389,564) | GAGTGGGGAAAGGGGAAGG | GTCTGTCGGTCGATCCTCC |
| *CTGF* | chr6 (132,272,959  -132,273,164) | GTCACACGCGTCTTTGTTCT | CCCCAACCCTTAGCAATGAT |
| *IFI16* | chr1 (159,009,400  -159,009,591) | GCAGACATTTCCCCAACCAG | ACCCAGCAGACTTACATGTGA |
| *IGLON5* | chr19 (51,814,661  -51,814,822) | AGTCTCCTAGGTCCATGGGA | GAGAGGCCTTTGTTACTGCG |
| *SLC43A2* | chr17 (1,531,983  -1,532,185) | GGCGGCAGTGTTTTCTTCTT | CCATCTCTCTCCTCGTTCCC |
| *GAPDH* | chr12 (6,643,153  -6,643,577) | TAGGCCTTTGCCTGAGCAGTCCGGTGT | TTGAGGCCTGAGCTACGTGCGCCCGTAA |
| **Gene** | | **Quantitative MSP** | |
|  |  | **Forward Primer (5’-3’)** | **Reverse Primer (5’-3’)** |
| *APBB2* | | ATTAGTTATACGGAGGGGATATTCG | ACAAATAAAAACGACAAAACCTACG |
| *CHGA* | | TGGACGATGTTAGTTAATGATAAATC | TTAAACTCCAACTCTAAAAACCGTA |
| *CTGF* | | AGTGTTAAGGGGTTAGGATTAATTC | AACGACCCGAAACTTTTATACG |
| *Alu* | | ATTAGTCGGGCGTGGTGG | CCCGAATTCAAACGATTCTCC |

| **Table S2. Signaling pathway of target genes by KEGG database.** | | |
| --- | --- | --- |
| **Gene** | **Accession No.** | **KEGG pathway** |
| *ANGPT1* | NM_001146.3; NM_001199859.1; NM_139290.1 | Ras signaling pathway [PATH:hsa04014]; Rap1 signaling pathway [PATH:hsa04015]; HIF-1 signaling pathway [PATH:hsa04066]; PI3K-Akt signaling pathway [PATH:hsa04151]; Rheumatoid arthritis [PATH:hsa05323] |
| *APBB2* | NM_001166050.1; NM_001166051.1; NM_001166052.1; NM_001166053.1; NM_001166054.1; NM_004307.1; NM_173075.4 | -- |
| *ATP5A1* | NM_001001935.2; NM_001001937.1; NM_001257334.1; NM_001257335.1; NM_004046.5 | Oxidative phosphorylation [PATH:hsa00190]; Alzheimer's disease [PATH:hsa05010]; Parkinson's disease [PATH:hsa05012]; Huntington's disease [PATH:hsa05016] |
| *C10orf140* | NM_207371.3 | -- |
| *C10orf4* | NM_145246.4; NM_203438.2; NM_203439.2; NM_203440.2; NM_203441.2 | -- |
| *CASC1* | NM_001082972.1; NM_001082973.1; NM_001204101.1; NM_001204102.1; NM_018272.3 | -- |
| *CHGA* | NM_001275.3 | -- |
| *CTGF* | NM_001901.2 | Hippo signaling pathway [PATH:hsa04390] |
| *HAUS1* | NM_138443.3 | -- |
| *IFI16* | NM_001206567.1; NM_005531.2 | -- |
| *IGLON5* | NM_001101372.1 | -- |
| *LBR* | NM_002296.3; NM_194442.2 | -- |
| *LMAN1* | NM_005570.3 | Protein processing in endoplasmic reticulum [PATH:hsa04141] |
| *LYRM5* | NM_001001660.2 | -- |
| *MCOLN1* | NM_020533.2 | Lysosome [PATH:hsa04142] |
| *MTMR8* | NM_017677.3 | -- |
| *PDZRN3* | NM_015009.1 | -- |
| *PLCL2* | NM_001144382.1; NM_015184.5 | -- |
| *PLEKHM2* | NM_015164.2 | Salmonella infection [PATH:hsa05132] |
| *RASAL1* | NM_001193520.1; NM_001193521.1; NM_004658.2 | -- |
| *SLC43A2* | NM_001284498.1; NM_001284499.1; NM_152346.2 | -- |
| *SP1* | NM_001251825.1; NM_003109.1; NM_138473.2 | TGF-beta signaling pathway [PATH:hsa04350]; Estrogen signaling pathway [PATH:hsa04915]; Transcriptional misregulation in cancers [PATH:hsa05202]; Huntington's disease [PATH:hsa05016] |
| *TCERG1L* | NM_174937.3 | -- |
| *ZNF175* | NM_007147.2 | -- |
| *ZNF69* | NM_021915.2 | -- |
| *ZSWIM5* | NM_020883.1 | -- |
